# A new species of *Petta* (Annelida, Pectinariidae), with comments on *Pettaassimilis* McIntosh, 1885

**DOI:** 10.3897/zookeys.1067.72596

**Published:** 2021-10-29

**Authors:** Jinghuai Zhang, Pat Hutchings

**Affiliations:** 1 South China Sea Environmental Monitoring Center, State Oceanic Administration, 155 Xingangxi Road, Guangzhou, China South China Sea Environmental Monitoring Center, State Oceanic Administration Guangzhou China; 2 Key Laboratory of Marine Environmental Survey Technology and Application, Ministry of Natural Resources, 353 Xingangxi Road, Guangzhou, China Key Laboratory of Marine Environmental Survey Technology and Application, Ministry of Natural Resources Guangzhou China; 3 Australian Museum Research Institute, Australian Museum, 1, William Street, 2010, New South Wales, Australia Australian Museum Research Institute, Australian Museum New South Wales Australia; 4 Department of Biological Sciences, Macquarie University, North Ryde, 2019, Australia Macquarie University North Ryde Australia

**Keywords:** Indian Ocean, new species, Polychaeta, Pectinariidae, taxonomy

## Abstract

The genus *Petta* Malmgren, 1866 is a small and poorly known genus of the annelid family Pectinariidae Quatrefages, 1866. A previous revision of the genus found that the type material of the species *P.assimilis* McIntosh, 1885 had been lost. While searching for material from the type locality, we were able to examine material from a similar area but collected in much shallower water from off South Africa which represents another undescribed species of *Petta*. The new species, *Pettabrevis***sp. nov.**, is described and compared to *P.assimilis* McIntosh, 1885, and a revised key to all species in the genus is provided.

## Introduction

Pectinariidae Quatrefages, 1866 is a small family of terebelliform polychaetes easily recognized by their characteristic ice cream cone shaped tubes made of sand grains cemented with mucus, and large opercular paleae. They typically inhabit soft sediments and use their buccal tentacles to sort organic particles in the sediment and carry them to the mouth (Hutchings 2000). Currently, this family includes five genera and 65 recognized species (Hutchings et al. 2021).

**Figure 1.**
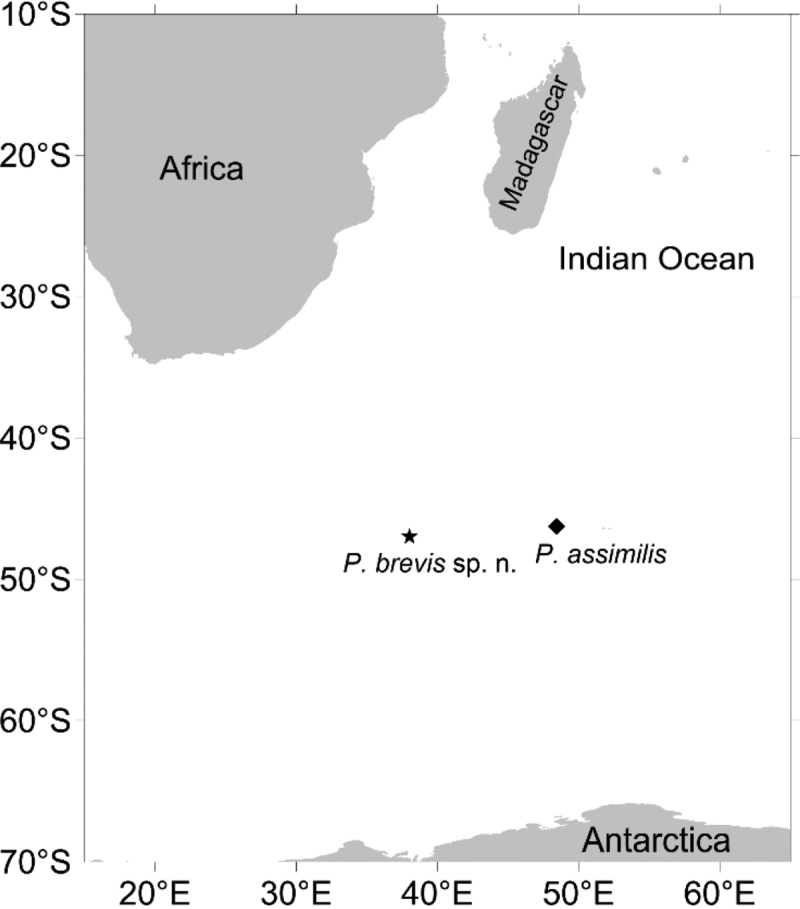
Type localities of *Pettabrevis* sp. nov. and *Pettaassimilis* McIntosh, 1885.

The genus *Petta* Malmgren, 1866 is characterised by having: cephalic veil completely free from the operculum, with the margins either smooth or bearing several lappets and having a pair of ear-shaped lobes adjacent to the dorsal side of the veil; operculum semi-circular with smooth dorsal and lateral margins and a traverse row of numerous stout paleae on the ventral margin; two pairs of comb-like branchiae; seventeen pairs of notopodia on segments 5–21, with capillaries, and neuropodia from segment 7 or 8; neurochaetae as avicular uncini, with crest with transverse rows of progressively shorter teeth; scaphe not clearly separated from posterior body segments, with six pairs of distinct triangular lobes on the lateral margins and a vestigial anal flap. Species of *Petta* are distinguished by the number of pairs of scaphal hooks, by the presence or absence of an anal cirrus, by the numbers and shape of ventro-lateral lobes on segment 2 and 3, and by the number of pairs of neuropodia (Nogueira et al. 2019; Zhang et al. 2019). A revision of the genus, consisting of six species, by Zhang et al. (2019) found that the holotype of *P.assimilis* McIntosh, 1885, collected of South Africa during the “Challenger Expedition” , which had been lodged in the Natural History Museum, London, had been lost (Muir, pers. comm.), so they just provided a brief description based on McIntosh’s original one. Subsequently, we had the opportunity to examine some material described by Branch (1994) as *P.assimilis*, collected from fairly close to the type locality south of South Africa (Fig. 1), but no detailed morphological description was provided. McIntosh described his single specimen from a depth of 2926 m, whereas Branch (1994) recorded the species at 360–376 m. Considering the limited data on the depth distributions of other species of *Petta*, there are no records of species occurring over such a depth range. The material reported by Branch (1994) and fixed in formalin, also has a different arrangement of anterior ventral pads, so we have described it as a new species, *P.brevis* sp. nov. Hopefully, additional material will be collected from a similar depth and location as that of *P.assimilis* so that a neotype can be designated at a later stage, as the original description lacks details regarding characters that Zhang et al. (2019) suggested are important for distinguishing species in this genus. Ideally, any additional material collected of *Pettabrevis* sp. nov. from the type locality will be fixed in a way that can be used for molecular studies.

## Material and methods

Five specimens were examined from 46°59'45"S, 38°00'39"E, at depths of 360–376 m, between Marion and Prince Edward Island south of South Africa. The holotype was stained with methyl green for photography using a Canon EOS 7D camera with a Macro EF 100 mm lens and a Spot Flex CCD 15.2 fitted on a Leica MZ16 Stereo microscope at the Australian Museum, Sydney. The software Helicon Focus 5.3 was used for focus stacking. Another specimen from the same sample was dehydrated in ethanol, critical point dried, coated with 20 nm of gold and examined under a JEOL JSM-6480 Scanning Electron Microscope (SEM) at Macquarie University, Sydney. Terminology follows that of Hutchings et al. (2019). Data on the holotype are given, with the variations of the other material, all designated as paratypes, given in parentheses in the case of complete specimens. All material is deposited in Iziko Museums of South Africa (formerly South African Museum, Cape Town).

## Results

### Petta brevis sp. nov.

Taxon classificationAnimaliaTerebellidaPectinariidae

98636BC9-3818-5F9B-9B27-FF8AF9C29D6B

http://zoobank.org/BFEB135F-5072-4841-B65B-0F31C5F512D7

Figs 1 , 2 , 3

Petta assimilis – Branch 1994: 15 (Prince Edward Island). – Zhang et al. 2019: 311–312. Not P.assimilis McIntosh, 1885

#### Type material.

***Holotype***: SAM A021260, from Station No MAD 39 FFF; 46°59'45"S, 38°00'39"E, depth 360–376 m, Marion and Prince Edward Island, South Africa, collected by bottom dredge, University Marion Island Survey, coll. 26 August 1987 by M. Branch.

***Paratypes***: SAMC A094445, 1 specimen complete, prepared for SEM, and 3 incomplete specimens, with parts of body wall dissected and empty tubes. All paratypes collected from same location as holotype. All material fixed in formalin and then transferred to 70% alcohol.

#### Etymology.

The specific epithet *brevis* is Latin for “shallow”, which refers to the type locality of new species, collected in relatively shallow waters compared to *Pettaassimilis*, which is known from a nearby area but in much deeper water.

#### Description.

**Figure 2.**
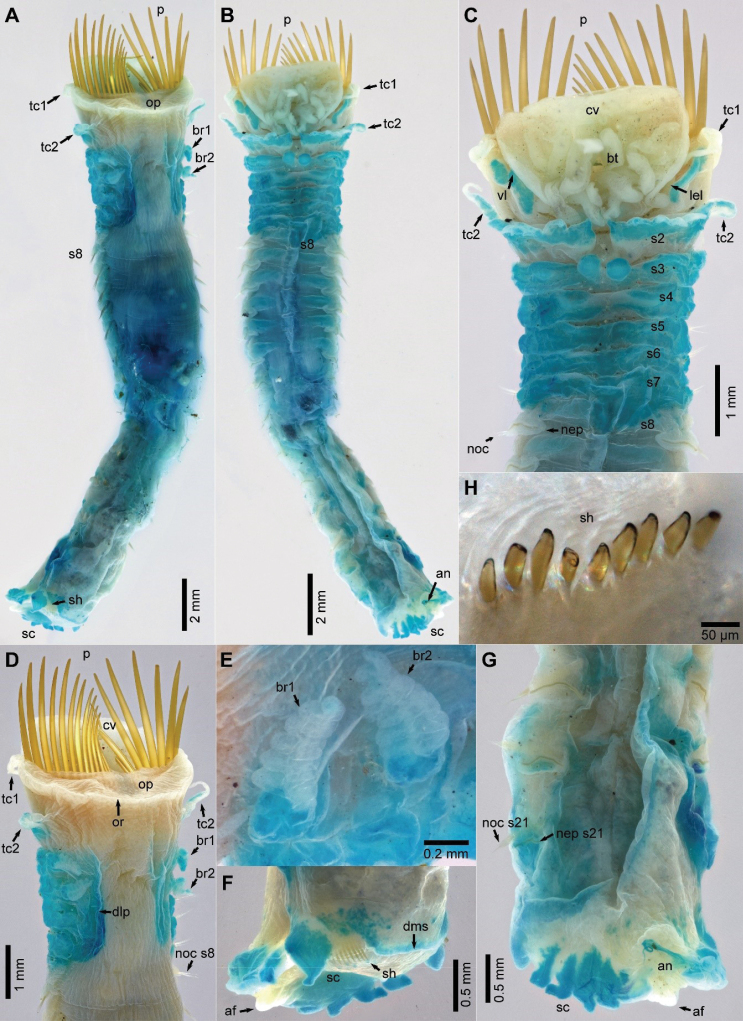
*Pettabrevis* sp. nov., holotype (SAM-A021260) **A** dorsal view of whole body **B** ventral view of whole body **C** ventral view of anterior end **D** dorsal view of anterior end **E** close up of left branchiae **F** dorso-lateral view of posterior end **G** ventral view of posterior end **H** close up of scaphal hooks. Abbreviations: af, anal flap (plate); an, anus; br, branchiae; bt, buccal tentacles; cv, cephalic veil; dlp, dorso-lateral pad; dms, dorsal margin of scaphe; lel, lateral ear-shaped lobe; nec, neurochaetae; nep, neuropodium; noc, notochaetae; op, operculum; or, opercular rim; p, paleae; s, segment; sc, scaphe; sh, scaphal hooks; tc, tentacular cirri; vl, ventral lappet.

**Figure 3.**
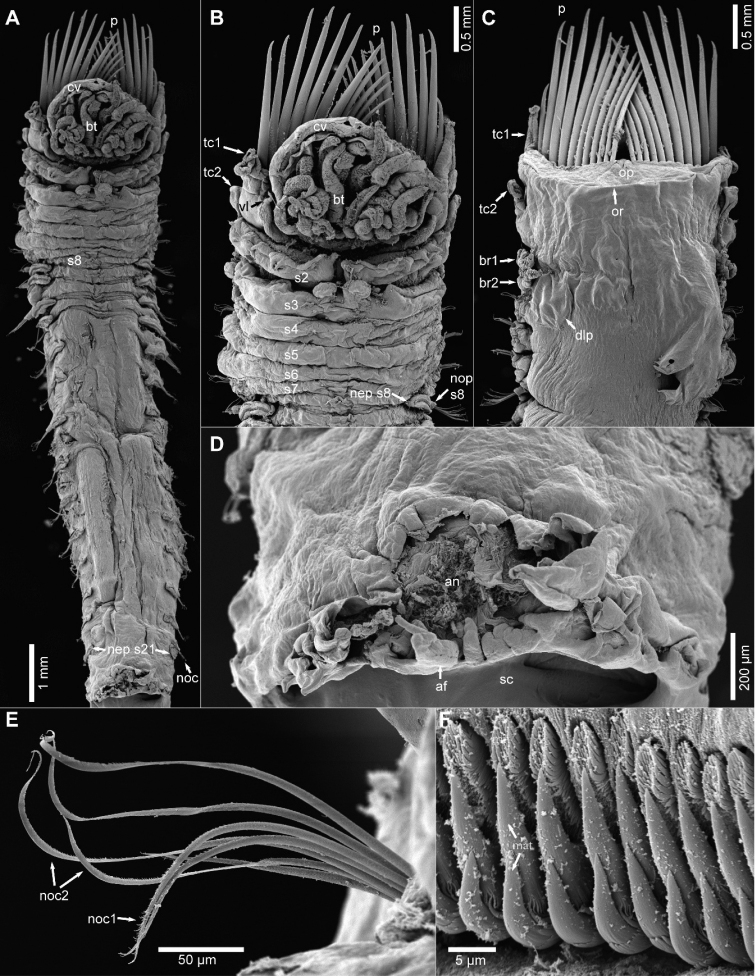
*Pettabrevis* sp. nov., SEM of paratype (SAMC-A094445) **A** ventral view of whole body **B** ventral view of anterior end **C** dorsal view of anterior end **D** ventral view of posterior end with distorted scaphe **E** notochaetae **F** close up of uncini. Abbreviations: af, anal flap (plate); an, anus; br, branchiae; bt, buccal tentacles; cv, cephalic veil; dlp, dorso-lateral pad; mat, major teeth; nec, neurochaetae; nep, neuropodium; noc, notochaetae; nop, notopodium; op, operculum; or, opercular rim; p, paleae; s, segment; sc, scaphe; tc, tentacular cirri; vl, ventral lappet.

Holotype pale in colour except for golden paleae and sand grains visible through the body wall. Body cylindrical, tapering before scaphe (Figs 2A, B, 3A). Length 15 mm (20) including paleae and scaphe, maximum width 3 mm (3).

Cephalic veil semi-circular, free from operculum, with smooth lateral margins, distal (anterior) end thin, folded over with smooth margins (Fig. 2A–C). Pair of lateral ear-shaped lobes adjacent to dorsal side of cephalic veil (Fig. 2C). Buccal tentacles numerous, thick, with deep longitudinal grooves arising around buccal cavity (Figs 2B, C, 3C).

Operculum semi-circular, surface tessellated and slightly inflated, with lateral and dorsal margins slightly elevated but smooth (Figs 2A, D, 3C). Two rows of 13 pairs of golden coloured paleae, some broken but arranged in a fan shape, differing slightly in length, outer ones shorter and thinner than inner ones on each row, slightly curved dorsally, with blunt tips (Figs 2C, D, 3C).

First pair of tentacular cirri extending to about halfway along the outer paleae, slightly annulated with swollen base tapering to long thin tip arising from base of opercular margin and paleal ridge (Figs 2A–D, 3B, C). Pair of blunt-tipped triangular ventral lappets present just laterally to first pair of tentacular cirri (Figs 2C, 3A, B).

Second pair of tentacular cirri similar in length to first pair but thinner and with base less swollen than that of first pair (Figs 2A–D, 3B, C), inserted slightly dorsally on mid lateral connecting ridge of segment 2. Segment 2 with pair of broad ventro-lateral lobes separated by a broad and deep mid-ventral groove; each lobe with six pairs of triangular lappets, two mid ventral ones largest (Figs 2B, C, 3B, C).

Segments 3 and 4 with two pairs of similar-sized branchiae, those of segment 3 slightly displaced ventrally (Figs 2A, D, 3C). Each branchia with large basal hump and with about six loose, flat lamellae (Fig. 2D, E). Segment 3 with raised ventral ridge with pair of broad ventro-lateral lobes and pair of rectangular mid-ventral lobes, mid-ventral lobes with rounded margins (Figs 2C, 3B). Segment 4 with slightly raised ventral margins with slight mid ventral indentation, but ventral margins less glandular than those of segment 3 (Figs 2B, C, 3A, B). Pair of dorso-lateral pads small and smooth, arising from dorsal side of notopodia on segment 5 (Figs 2D, 3C), but distorted on segment 5 of holotype.

Discrete raised ventral glandular lobes (pads) on segments 2–7, decreasing in size and elevation posteriorly with slight mid-ventral indentation (Figs 2C, 3B).

Notopodia of segment 1 with paleae, and notochaetae from segments 5–21 (17 pairs). Notopodia of segments 5–7 smaller and with smaller notochaetae (Figs 2D, 3C); notopodia of segments 8–13 relatively large with long chaetae; following notopodia decreasing posteriorly in size, length of notochaetae similarly decreasing. Notopodia with 2 rows of different capillary notochaetae; one with distal serrated wings, anterior surface covered with numerous minute spines from below wing to about mid-basal portion of chaeta; another tapering to acute tip without wings, anterior surface covered with numerous spines from mid-length to tip (Fig. 3E).

Neuropodia developed from segment 8 (Figs 2B, C, 3A, B), continuing to scaphal plate, with slightly raised tori. Neurochaetae (uncini) arranged in single transverse row on each torus. Uncinus with two main teeth, followed by several rows of numerous small teeth (Fig. 3F) and a large peg with blunt tip embedded into torus. Neuropodia of segment 21 with enlarged posterior lobe (Fig. 3A).

Scaphe long, ovoid, flattened dorsally, inconspicuous constriction on posterior segments. Lateral margins rolled dorsally, with six pairs of lobes; first pair largest, connected to dorsal margin of scaphe; posterior lobes narrow, triangular, almost of equal size; dorsal margin of scaphe smooth (Figs 2F, G, 3D). Anal flap triangular, without an anal cirrus. Scaphal hooks amber-coloured, left 9 and right 10 on holotype, arising from both sides of dorsal margin of scaphe, with blunt tips slightly curved dorsally (Fig. 2H).

Type of tube: with thin chitinous inner lining covered in small stones cemented together.

#### Variation.

The paratypes consist of one complete specimen, prepared for SEM, and three anterior fragments with some parts of their body wall dissected and some empty incomplete tubes.

#### Remarks.

*Pettabrevis* sp. nov. is characterised by a cephalic veil with smooth margins, 13 pairs of paleae, 2 pairs of similar length tentacular cirri, segment 2 with pair of broad ventro-lateral lobes, each lobe with six pairs of triangular lappets, segment 3 with pair of ventro-lateral lobes, and rectangular mid ventral lobes, elongate scaphe not well separated from posterior body, lateral margin with six pairs of lobes, 9–10 pairs of blunt tipped scaphal hooks and smooth anal flap.

*Pettabrevis* sp. nov. differs from *P.pusilla* Malmgren, 1866, which has the anterior margin of the cephalic veil with several lappets, in having a smooth margin to the cephalic veil, like all other species of *Petta*. The arrangement of lobes on segment 3 in *Pettabrevis* sp. nov. differentiates it from *P.assimilis* McIntosh, 1885 and *P.investigatoris* Zhang, Hutchings & Kupriyanova, 2019, as these two species have lappets on the ventral lateral lobes of segment 3. The number of pairs of scaphal hooks also differs between species as well as the presence or absence of an anal cirrus: *P.pusilla* has 8 pairs of scaphal hooks and anal cirrus present; *P.pellucida* has 7 pairs of scaphal hooks and the presence/absence of anal cirrus was not stated; *P.tenuis* has 8 pairs of scaphal hooks and a long anal cirrus; *P.investigatoris* Zhang, Hutchings & Kupriyanova, 2019 and *P.williamsonae* Zhang, Hutchings & Kupriyanova, 2019 both have 9 pairs of scaphal hooks and long anal cirrus. For *P.assimilis*, no data on the number of pairs of hooks or whether an anal cirrus are present or not, was given, whereas *P.brevis* sp. nov. has 9 scaphal hooks on one side and 10 on the other and lacks an anal cirrus.

#### Discussion.

McIntosh described *P.assimilis* from Station 147 (between Prince Edward and Kerguelen Islands), 46°16'S, 48°27'E, at a depth of 1600 fathoms (= 2926 m), and recorded the sediment as being diatom ooze. His description was focussed on how similar the new species was to the British representative of the genus, which he did not actually name, and he also compared the new species with *P.pusilla* Malmgren, 1866, which was described from the west coast of Sweden (Zhang et al. 2019). As no other species had been described from Europe when McIntosh described his species, one must assume that he was referring to an undescribed English species. McIntosh illustrated the ventral view and the scaphal plate (McIntosh 1885, plate XLVII, figs 8, 9) and the chaetae (plate XXVIA, figs 16–19) in a schematic way but gave no information as to the number of pairs of scaphal hooks present. Branch (1994) also collected a species of *Petta* from a similar area but in much shallower water and identified it as *P.assimilis*. As noted above, we found differences between the material collected by Branch, and therefore described it as a new species. Also, the review of the genus by Zhang et al. (2019) found that all species of *Petta* have very restricted depth ranges, which also supports our conclusion that *Pettabrevis* sp. nov., although collected from a similar location as *P.assimilis* but at a much shallower depth, should be described as new.

Hartman (1967) also recorded *Pettaassimilis* from off Cape Horn (1806–2013 m) and the Falkland Islands, 2452 m, South America. Comparing her description with that of McIntosh (1885), it is difficult to decide if Hartman’s species is the same or yet another undescribed species of *Petta*. Certainly, the species recorded by Hartman differs from *Pettabrevis* sp. nov. in terms of number of pairs of paleae and the structure of the branchiae. Although she stated that three pairs are present, the first is actually the second tentacular cirrus, and the scaphe has six pairs of triangular lobes, anal cirrus present, and 11 pairs of caudal spines that are distally slightly falcate. She mentioned that the anterior margins of segments 2 and 3 have 7 pairs of fimbriae, which on the figure (Hartman 1967, fig. 44A) equate to the ventro-lateral lobes. We suggest that the identity of Hartman’s material cannot be determined at this stage. We also regard that *Pettaassimilis* is an indeterminate species until material from much closer to the type locality becomes available and can be examined.

This study supports the findings of Zhang et al. (2019) that species of *Petta* are not common and are mainly reported from the deep sea, with some species only known from type material. It must be noted that deep sea habitats are relatively poorly sampled around the world; however, because tubes of pectinariids are very conspicuous, one would expect them to be recorded if present, which reinforces our belief that this family is not well represented in the deep sea. The only exception is *P.pusilla*Malmgren 1866, which has been recorded from many locations at depths of 15–200 m (see Zhang et al. 2019 for a complete list); however, we suggest that these records should be checked, as its geographical range is very large with varying ecological conditions. Also, the holotype, which was examined by Zhang et al. (2019), differs from that given by Malmgren, which may have contributed to these widespread records.

### Taxonomic key to genera of Pectinariidae and to all species of the genus *Petta* (modified from Zhang et al. 2019)

**Table:** 

1	Opercular rim with cirri or lappets	***Amphictene***
–	Opercular rim smooth	**2**
2	Cephalic veil attached to lateral margin of operculum	***Lagis***
–	Cephalic veil free from operculum	**3**
3	More than one longitudinal row of major teeth on uncini	***Pectinaria***
–	One longitudinal row of major teeth on uncini	**4**
4	Lateral and anterior margins of cephalic veil with numerous cirri or lappets; anal flap present; pair of dorso-lateral pads absent on segment 5	***Cistenides***
–	Lateral and anterior margins of cephalic veil smooth or only anterior margin with several lappets; anal flap vestigial; pair of dorso-lateral pads present on segment 5	***Petta* 5**
5	Anterior margin of cephalic veil with several lappets	***P.pusilla* Malmgren, 1866**
–	Anterior margin of cephalic veil smooth	**6**
6	Ventro-lateral lobes with continuous row of lappets on segment 3	**7**
–	Ventro-lateral lobes smooth, without lappets on segment 3	**8**
7	Anal flap without anal cirrus; ventro-lateral lobes with 4–5 lappets on segment 2	***P.assimilis* McIntosh, 1885**
–	Anal flap with long anal cirrus; ventro-lateral lobes with 7–8 lappets on segment 2	***P.investigatoris* Zhang, Hutchings & Kupriyanova, 2019**
8	Scaphe distinctly separated by a constriction from posterior segments	***P.williamsonae* Zhang, Hutchings & Kupriyanova, 2019**
–	Scaphe not separated by a constriction from posterior segments	**9**
9	Scaphal hooks 5–8	**10**
–	Scaphal hooks more than 9	**11**
10	Scaphal hooks 7; lobes of segment 2 with pair of ventralmost cirri distinctly longer than other cirri; longer mid-ventral lobes on segment 3, cylindrical and distally rounded; neuropodia on segments 7–21	***P.pellucida* (Ehlers, 1887)**
–	Scaphal hooks 5–8; cirri of lobes of segment 2 are all of even length; the mid-ventral lobes of segment 3 are spherical; neuropodia on segments 8–21	***Pettaalissoni* Nogueira, Ribeiro, Carrerette & Hutchings, 2019**
11	Ventro-lateral lobes with 4–5 lappets on segment 2; scaphal hooks 11	***P.tenuis* Caullery, 1944**
–	Ventro-lateral lobes with 6 lappets on segment 2; scaphal hooks 9–10	***P.brevis* sp. nov.**


## Supplementary Material

XML Treatment for Petta brevis
